# Beginning at the ends: telomeres and human disease

**DOI:** 10.12688/f1000research.14068.1

**Published:** 2018-05-01

**Authors:** Sharon A. Savage

**Affiliations:** 1 Clinical Genetics Branch, Division of Cancer Epidemiology and Genetics, National Cancer Institute, Bethesda, Maryland, USA

**Keywords:** telomere, dyskeratosis congenita, telomere biology disorder, cancer, Coats plus, Hoyeraal Hreidsarsson syndrome, epidemiology

## Abstract

Studies of rare and common illnesses have led to remarkable progress in the understanding of the role of telomeres (nucleoprotein complexes at chromosome ends essential for chromosomal integrity) in human disease. Telomere biology disorders encompass a growing spectrum of conditions caused by rare pathogenic germline variants in genes encoding essential aspects of telomere function. Dyskeratosis congenita, a disorder at the severe end of this spectrum, typically presents in childhood with the classic triad of abnormal skin pigmentation, nail dystrophy, and oral leukoplakia, accompanied by a very high risk of bone marrow failure, cancer, pulmonary fibrosis, and other medical problems. In contrast, the less severe end of the telomere biology disorder spectrum consists of middle-age or older adults with just one feature typically seen in dyskeratosis congenita, such as pulmonary fibrosis or bone marrow failure. In the common disease realm, large-scale molecular epidemiology studies have discovered novel associations between illnesses, such as cancer, heart disease, and mental health, and both telomere length and common genetic variants in telomere biology genes. This review highlights recent findings of telomere biology in human disease from both the rare and common disease perspectives. Multi-disciplinary collaborations between clinicians, basic scientists, and epidemiologist are essential as we seek to incorporate new telomere biology discoveries to improve health outcomes.

## Introduction

Telomeres are the nucleoprotein complex at chromosome ends with essential roles in maintaining chromosomal integrity. They shorten with each cell division because of incomplete replication of the 3′ ends of DNA and thus are markers of cellular aging. Over the last decade, there has been remarkable growth in the breadth and depth of understanding the multiple roles of telomere biology in human disease. At one end of the spectrum, very rare pathogenic germline genetic variants in telomere biology genes cause exceedingly short telomeres, resulting in dyskeratosis congenita (DC) and its related telomere biology disorders. The other end of the spectrum consists of large-scale population-based studies seeking to determine associations between telomere length human disease, environmental exposures, or common genetic variants as well as the interactions between these factors.

**Figure 1.**
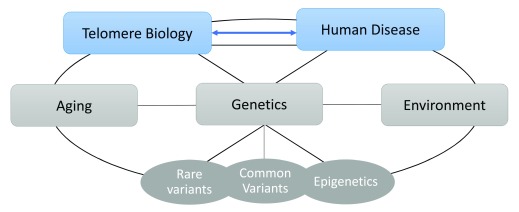
Factors associated with human disease are integrally connected to telomere biology. This schematic illustrates the complex relationships between telomere biology, disease, aging, genetics, and environmental exposures, all of which should be considered in studies of telomeres and human disease.

The complexity of these interactions requires an integrated understanding of telomere basic science, clinical medicine, and epidemiology ( Figure 1). Each of these topics is worthy of an in-depth critical review beyond the scope of this article. Instead, I will highlight some key findings and methodologic considerations and discuss where additional research is needed to aid in understanding the contribution of telomere biology to both rare and common human diseases.

## Dyskeratosis congenita – a direct connection between germline telomere biology and human disease

**Figure 2.**
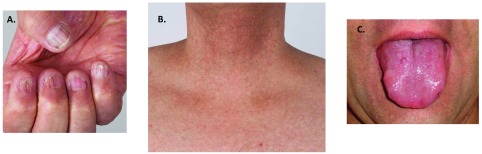
Mucocutaneous features of dyskeratosis congenita of an adult male whose disease is due to a *DKC1* mutation. ( **A**) Dystrophic and ridged fingernails. ( **B**) Hyper- and hypo-pigmented skin of neck and upper chest. ( **C**) Irregular leukoplakia of the tongue.

DC was first described in a 1906 case report of males with the mucocutaneous triad of abnormal skin pigmentation, nail dystrophy, and oral leukoplakia ( Figure 2) ^1^. Additional similar cases were reported, including the first female case in 1963 ^2– 5^. Patients with DC also have very high rates of bone marrow failure; stenosis of the esophagus, urethra, or lacrimal ducts (or a combination of these); head and neck squamous cell carcinoma (HNSCC); myelodysplastic syndrome (MDS); acute myeloid leukemia (AML); pulmonary fibrosis; liver disease; avascular necrosis of the hips; and other medical problems ( Table 1) ^6^. DC is inherited in X-linked recessive, autosomal dominant, or autosomal recessive patterns.

**Table 1.** Clinical features of telomere biology disorders.

Disorder	Key clinical features ^a^
Dyskeratosis congenita (DC)	Mucocutaneous triad of nail dysplasia, abnormal skin pigmentation (hyper/hypopigmented, lacy, reticular pigmentation), and oral leukoplakia. BMF, PF, PAVM, liver disease, avascular necrosis of hips or shoulders (or both), urethral stenosis, lacrimal duct stenosis, esophageal stenosis, HNSCC, MDS, AML, and/or developmental delay. Traditional diagnosis of DC: classic triad *or* one of the triad, BMF, and two other findings ^7^.
Revesz syndrome	Features of DC plus bilateral exudative retinopathy. Intracranial calcifications and developmental delay also reported.
Hoyeraal Hreidarsson syndrome	Features of DC plus cerebellar hypoplasia. Immunodeficiency has been reported as presenting problem.
Coats plus	Bilateral retinopathy, intracranial calcifications, leukodystrophy, anemia, osteopenia, and poor bone healing
DC-like	BMF, AA, MDS, or PF occurring in presence of at least one other DC-associated feature or family history suspicious of DC
Aplastic anemia	Progressive multi-lineage cytopenias, non-immune mediated. May occur in the absence of DC- associated features.
Myelodysplastic syndrome	Cytopenias with cellular dysplasia or clonal chromosomal translocations or both. May occur in the absence of DC-associated features.
Acute myeloid leukemia	May progress from MDS or aplastic anemia. May occur in the absence of DC-associated features.
Pulmonary fibrosis	May occur in the absence of DC-associated features.
Liver fibrosis	Non-alcoholic, non-infectious liver disease. May occur in the absence of DC-associated features.
Familial melanoma	Multiple family members with melanoma, often early age at onset
Familial lymphoproliferative disease	Multiple-affected family members with chronic lymphocytic leukemia, or non-Hodgkin lymphoma
Li-Fraumeni-like syndrome	Cancer family history notable for angiosarcoma and other cancers

^a^Key references are noted in Table 2. AA, aplastic anemia; AML, acute myeloid leukemia; BMF, bone marrow failure; DC, dyskeratosis congenital; HNSCC, head and neck squamous cell carcinoma; MDS, myelodysplastic syndrome; PAVM, pulmonary arteriovenous malformation; PF, pulmonary fibrosis.

**Figure 3.**
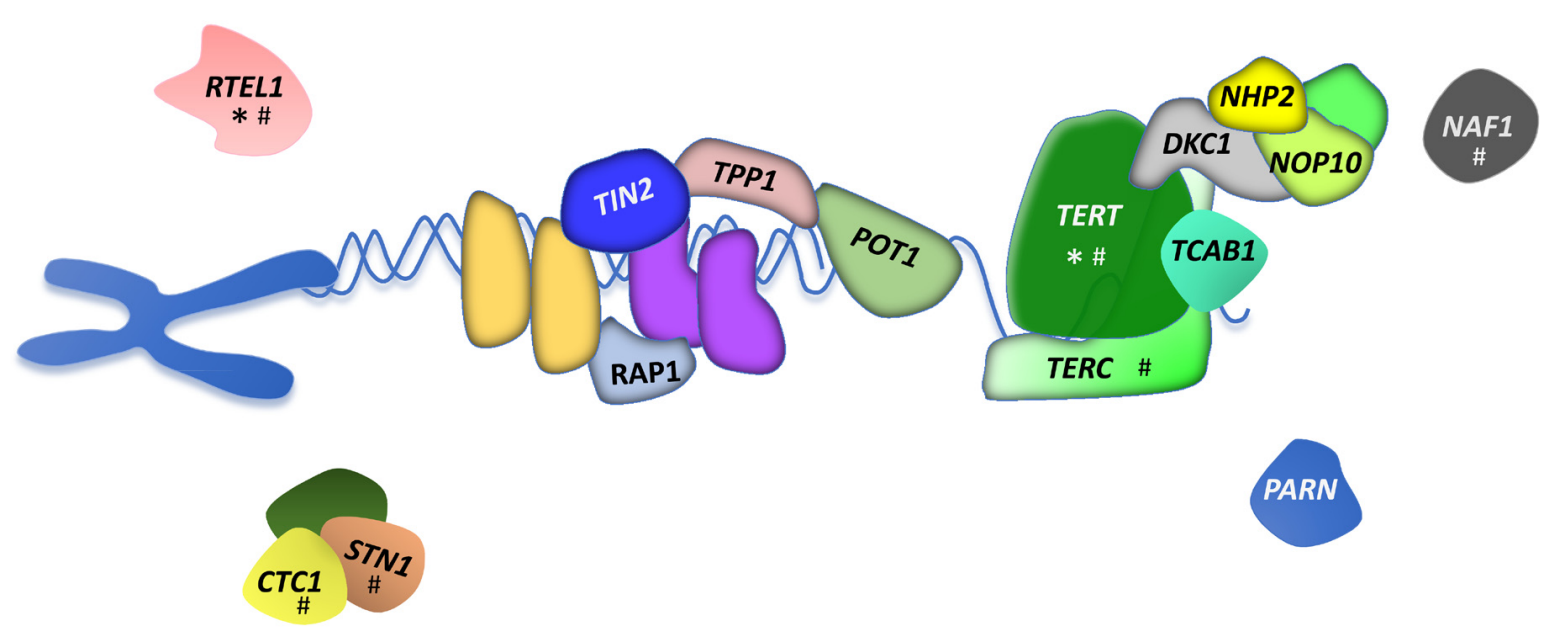
Proteins encoding key components of telomere biology associated with disease. Protein names are noted in the figure. Associated disease and mode(s) of inheritance are shown in Table 1. The asterisk indicates proteins encoded by genes with single-nucleotide polymorphisms associated with cancer. The pound sign indicates proteins encoded by genes with single-nucleotide polymorphisms associated with telomere length.

The first DC genetic locus was mapped to Xq28 in 1996 and specifically to mutations in dyskerin (encoded by *DKC1*) in 1999 ^8– 10^. The seminal work by Mitchell and Collins was the first to show a connection between telomere biology and human disease through aberrant dyskerin function and the resultant very short telomeres now well known in patients with DC ^11^. Currently, *DKC1* mutations account for about 25% of classic DC cases. A combination of candidate gene sequencing, genetic linkage studies, and whole exome sequencing occurring over the last 15 years has since identified at least 14 telomere biology genes associated with DC or DC-like phenotypes: telomerase holoenzyme complex ( *DKC1*, *TERC*, *TERT*, *NOP10*, and *NHP2*), shelterin telomere protection complex ( *ACD*, *TINF2*, and *POT1*), telomere capping proteins ( *CTC1* and *STN1*), and other proteins that directly or indirectly interact with these key cellular processes ( *RTEL1*, *NAF1*, *WRAP53*, and *PARN*) ( Figure 3 and Table 2) (reviewed in 6).

**Table 2.** Germline genetics of telomere biology disorders.

Gene	Protein name(s)	Disorder(s)	Mode of inheritance	Year gene first associated with any disease, relevant reference(s)
*DKC1*	DKC1, dyskerin	DC, HH	XLR	1998 ^23^
*TERC*	hTr, telomerase RNA component (encodes an RNA)	DC, AA, PF	AD	2001 ^16, 18, 24^
*TERT*	TERT, telomerase	DC, AA, MDS, AML, PF, LD, FM	AD	2005 ^16, 17, 25– 27^
		HH	AR	
*NOP10*	NOP10, NOLA3, nucleolar protein family A, member 3	DC	AR	2007 ^28^
*NHP2*	NHP2, NOLA2 nucleolar protein family A, member 2	DC	AR	2008 ^29^
*TINF2*	TIN2, TERF1 (TRF1)-interacting nuclear factor 2	DC, HH, RS	AD	2008 ^15^
*WRAP53*	TCAB1, telomere cajal body associated protein 1	DC, HH	AR	2011 ^30, 31^
*CTC1*	CTC1, conserved telomere maintenance component 1	CP, DC	AR	2012 ^32– 34^
*RTEL1*	RTEL1, regulator of telomere elongation helicase 1	PF, AA	AD	2013 ^35– 40^
		DC, HH	AR	
*TERF2IP*	RAP1, TERF2-interacting protein	FM	AD	2015 ^41^
*PARN*	PARN, poly(A)-specific ribonuclease	PF	AD	2015 ^42– 45^
		DC, HH	AR	
*ACD*	TPP1, telomere protection protein 1	AA, FM, FLPD	AD	2014 ^41, 46, 47^
		HH	AR	2016 ^48^
*STN1*	STN1, CST-complex subunit	CP	AR	2016 ^49^
*POT1*	POT1, protection of telomeres 1	FM, FLPD, LFL	AD	2014 ^47, 50– 52^
		CP	AR	2016 ^53^
*NAF1*	NAF1, nuclear assembly factor 1 ribonucleoprotein	PF	AD	2016 ^54^

AA, aplastic anemia; AD, autosomal dominant; AML, acute myeloid leukemia; AR, autosomal recessive; CP, Coats plus; DC, dyskeratosis congenita; FLPD, familial lymphoproliferative disease; FM, familial melanoma; HH, Hoyeraal-Hreidarsson syndrome; LD, liver disease; LFL, Li-Fraumeni-like syndrome; MDS, myelodysplastic syndrome; PF, pulmonary fibrosis; RS, Revesz syndrome; XLR, X-linked recessive.

**Figure 4.**
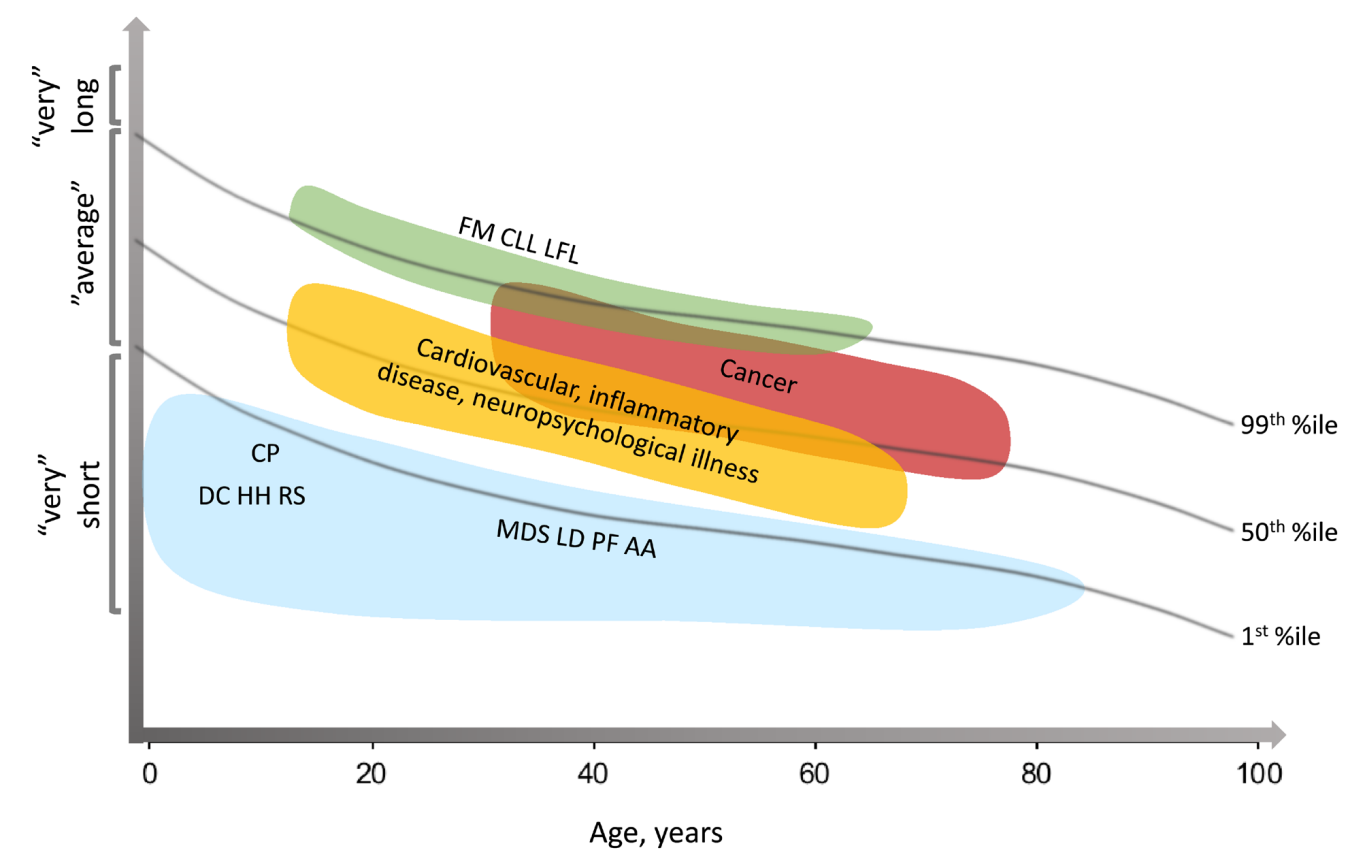
Schematic representation of the connections between age, telomere length, and human disease. Clinically significant telomeres associated with telomere biology disorders are generally at or below the first percentile for age (blue shape). Many association studies of telomere length and human phenotypes, including cancer, have identified statistically significant, but perhaps not clinically significant, differences in telomere length between cases and controls (represented by yellow and red). Some studies have identified rare families with germline mutations in components of the shelterin telomere protection complex as associated with longer telomeres (green shape). AA, aplastic anemia; CLL, chronic lymphocytic leukemia; CP, Coats plus; DC, dyskeratosis congenita; FM, familial melanoma; HH, Hoyeraal Hreidarsson syndrome; LD, liver disease; LFL, Li-Fraumeni-like syndrome; MDS, myelodysplastic syndrome; PF, pulmonary fibrosis; RS, Revesz syndrome.

The germline mutations in DC-associated telomere biology genes result in very short telomere lengths for age ( Figure 4). This knowledge made it possible to develop a diagnostic test for DC, flow cytometry with fluorescent *in situ* hybridization (flow FISH), in leukocyte subsets ^12^. Lymphocyte telomeres less than the first percentile for age are more than 95% sensitive and specific for differentiating patients with DC from their unaffected relatives or patients with other inherited bone marrow failure syndromes ^13, 14^. In addition to aiding diagnosis, using telomeres less than the first percentile for age has greatly added in discovering the genetic causes of DC ^15^.

## Telomere biology disorders – many names connected by pathophysiology

The discovery of the multiple genetic causes and modes of inheritance has led to a growing appreciation that there is a wide range of clinical phenotypes associated with mutations in telomere biology genes. This started with the identification of *TERT* and *TERC* mutations in patients with apparently isolated aplastic anemia or pulmonary fibrosis ^16– 18^. As defined above, classic DC is a complex multi-system disorder, but variable penetrance and expressivity of the clinical manifestations have identified a growing number of patients with one or a few features of DC as well as germline mutations in telomere biology genes and short telomeres ( Table 1 and Table 2). This spectrum of illnesses has been termed telomeropathy, short telomere syndromes, or telomere biology disorders (TBDs) ^6, 19– 22^. The last of these, TBD, was proposed and is favored because it is more descriptive and reflective of the underlying biology that unites these disorders ^6, 22^.

The most complex TBDs are those disorders presenting very early in childhood, namely Hoyeraal Hreidarsson syndrome (HH), Revesz syndrome (RS), and Coats plus ^6, 55– 60^. In addition to having features of DC, patients with HH have cerebellar hypoplasia and immunodeficiency, whereas those with RS also have bilateral exudative retinopathy. Coats plus, a disorder characterized by retinal and gastrointestinal vascular abnormalities, poor bone healing, leukodystrophy, and cerebellar calcifications, joined the TBD spectrum when its primary cause was identified as autosomal recessive *CTC1* mutations ^32, 33, 59^.

The other end of the clinical spectrum includes patients with middle or later age at onset of pulmonary fibrosis, liver disease, or bone marrow failure and heterozygous germline mutations in *NAF1*, *TERT*, *TERC*, *PARN*, or *RTEL1* ^17, 18, 42, 61^. ** Additionally, it is important to note that most patients do not have all of the DC-associated medical complications. The mucocutaneous triad is diagnostic but varies with the age of onset and is usually progressive over time. Many patients, even members of the same family, may present with just one feature but develop more over time because of variable penetrance and expressivity of germline telomere biology defects.

There is also a growing role of mutations in the shelterin complex and cancer-prone families without DC-related clinical manifestations. Heterozygous rare, pathogenic variants in *POT1* resulting in longer telomeres have been reported in familial melanoma, familial chronic lymphocytic leukemia (CLL), and a Li-Fraumeni-like syndrome family ^50– 52^. *POT1 s*omatic mutations in CLL have also been associated with CLL outcomes ^62– 65^. Familial melanoma has also been associated with germline mutations in *ACD* (TPP1) ** and *TERF2IP* (RAP1) ^41^. These studies suggest an interesting dichotomy in clinical phenotypes resulting from long versus short telomeres.

## Telomeres and cancer

Telomeres are closely connected to cancer biology because of the role they play in chromosomal stability. There is a detailed body of work in this realm and only a few of the key features will be highlighted herein as they have been reviewed in detail elsewhere ^66– 73^.

The primary hypotheses connecting telomeres and cancer are based on the fact that telomeres shorten with each cell division ^74^. In general, cellular senescence or apoptosis is triggered when telomeres reach a critically short state. It likely takes just one critically short telomere on one chromosome arm to trigger these events as suggested in a *TERC* mouse model by Hemann *et al*. ^75^. A cellular survival advantage is gained through bypassing apoptosis or senescence through the upregulation of telomerase, inactivation of TP53 or RB or both, initiation of alternative lengthening of telomeres (ALT), and other key biological pathways ^76– 80^. The continued division of cells originally destined for death is hypothesized to lead to continued accumulation of mutations, and sticky chromosome ends due to abnormal telomeres can contribute to chromosomal aneuploidy. Unchecked cellular growth can occur if these genetic aberrations result in a growth advantage.

Activating somatic mutations in the *TERT* promoter have been described in melanoma, bladder, thyroid, and some central nervous system cancers ^81– 83^. These somatic mutations in the *TERT* promoter result in increased telomerase expression and suggest that this activation could convey a growth advantage as cancer cells continue to divide despite the presence of aberrant telomeres.

Patients with DC/TBD have significantly increased risks of MDS, AML, HNSCC, and other malignancies ^84– 86^. The 2017 update of cancer in the National Cancer Institute Inherited Bone Marrow Failure Syndrome cohort reported that cancer in patients with DC occurs at an approximately four fold higher incidence and a younger age than the general population ^87^. MDS and AML occurred at 578- and 24-fold greater incidence, respectively, than the general population. There was also an excess of solid cancers in patients with DC with observed/expected ratios of 74 for any HNSCC and 216 for tongue HNSCC ^87^. The mechanisms by which cancer develops in patients with DC/TBDs is unknown and represents an important research opportunity. Cells of patients with TBD already have a “first hit” in a key component of telomere biology. Studies of the next steps in carcinogenesis in patient-derived cells could lead to important insights into the carcinogenic process.

## The advent of telomere molecular epidemiology

Telomere molecular epidemiology has emerged with the development of high-throughput telomere length measurement methods, genome-wide genotyping platforms, and keen interest in the role of telomere biology in human disease ^88, 89^. These large, often population-based studies seek to determine (1) whether telomere length is associated with disease, (2) whether common genetic variants (that is, ** single-nucleotide polymorphisms, or SNPs) are associated with telomere length, (3) the degree to which SNPs contribute to telomere biology, and (4) interactions between telomere length, SNPs, and disease or phenotypes ( Figure 1 and Table 3). Although a great deal of excitement has been generated by these studies, it is important to point out that differences in telomere length between cases and controls in large population-based studies may be statistically significant but not clinically relevant. “Short telomeres” in a large case-control or cohort study are still within the clinically “normal” range and not nearly as short as telomeres of patients with TBDs ( Figure 4).

**Table 3.** Features of robust telomere length association studies.

• Strong *a priori* hypothesis of why telomere biology might be important in disease of interest • Comprehensive clinical phenotyping • Accurately measured exposure of interest • Large sample size with power calculations reported • Collection of samples prior to disease onset • Detailed information on how samples were collected, processed, and stored • Telomere length measurement methods described in detail, especially if any adaptations to published methods • Accurate and reproducible telomere length measurement • Strong statistical justification of association findings

Robust and accurate telomere length measurement is at the crux of all telomere length association studies. Blood or buccal cell DNA telomere length has been evaluated in a wide array of association studies, including cancer, cardiovascular disease, mental health, inflammatory diseases, environmental exposures, and many other settings. There are numerous methods to determine telomere length in tissues, single cells, and DNA preparations, each appropriate for different applications and reviewed extensively ^90, 91^. Quantitative polymerase chain reaction (qPCR) is amenable to large studies because it uses very small amounts of DNA and can be scaled up rapidly ^92, 93^. However, qPCR telomere assays generate a relative telomere length and are very sensitive to DNA extraction methods and storage ^94^. These challenges have led to significant challenges in reproducing data in case-control or cohort studies of qPCR relative telomere length and phenotypes ^95, 96^. The telomere restriction fragment method uses restriction enzymes to cut the subtelomeric ends of chromosomes in a DNA preparation and is most widely used in basic science laboratories, although a few groups use it in population-based studies ^90, 91^.

### Blood or buccal cell telomere length association studies

This section highlights just a few key topics within the growing literature of telomere length association studies. For example, individuals of African ancestry have longer telomeres than those of European ancestry and thus ancestry should be accounted for in analyses ^97, 98^. Since self-reported ancestry can be quite variable, genomic approaches may be helpful in classifying cases and controls in order to appropriately adjust for ancestry.

There is also a growing understanding of associations between environmental exposures and telomere length. Smokers have shorter telomeres than non-smokers and thus it is important to adjust for smoking in association analyses ^99^. Associations between prenatal exposures to smoking and air pollution as well as exposure to certain occupational chemicals have also been explored but with varying results ^100– 105^. In each of these studies, it is essential to precisely quantify the exposure of interest in addition to using a robust and reproducible telomere length measurement.

Early cancer-telomere length association studies suggested shorter telomeres as a cancer risk factor ^106, 107^ but studies of other cancers were not consistent ^108– 112^. Meta-analyses found that most studies with blood or buccal cell DNA collected prior to cancer diagnosis were null but that case-control studies were more likely to find associations ^113, 114^. Similarly, a direct comparison of prospectively and retrospectively collected DNA samples from patients with breast or colorectal cancer reported that telomere shortening occurred primarily after cancer diagnosis ^115^. Many of these inconsistencies have been attributed to reverse causation bias due to the presence of cancer, underlying inflammation, or prior therapy at the time of sample collection ^113, 116^. Currently, the most consistent studies are those of longer telomeres in pre-diagnostic samples of patients with lung cancer and melanoma ^117– 119^. Interestingly, shorter leukocyte telomeres were associated with overall cancer mortality but not with cancer in a large prospective study of 64,637 individuals who developed 2,420 cancers ^120^.

There is a growing body of telomere length association studies and different aspects of mental health, including measures of perceived stress in caregivers, exposure to early life adversity, and in patients with schizophrenia, bipolar disorder, and depression ^121– 126^. The biological mechanisms underlying these findings are unknown but current hypotheses include stress responses inducing oxidative stress, resulting in DNA damage and telomere shortening. Notably, abnormalities in brain development are present in patients with HH (cerebellar hypoplasia), RS (intracranial calcifications), and Coats plus (leukodystrophy and intracranial calcifications) ^6, 55^. The only study to date of neuropsychiatric complications in DC found higher-than-expected occurrence of developmental delay and psychiatric disorders ^127^. Studies of DC/TBD patients by psychiatrists and neurobiologists constitute an unstudied area highly likely to generate important insights into telomere biology and brain development.

There is also a great deal of interest in using telomere length as a measure of biological age and even in modulating telomere length through lifestyle interventions. Numerous studies suggest associations between lifestyle, exercise, and telomere lengths ^128– 133^. However, a recent review suggests that telomere length in and of itself is not sufficient as a specific aging biomarker ^134^.

Current data consistently report shorter leukocyte telomeres in individuals with atherosclerotic cardiovascular disease than in unaffected controls ^135– 137^. The biology underlying this association is thought to be related to chronic inflammation and oxidative stress coupled with aging of the vasculature. This prompted Aviv *et al*. to propose a model whereby age-dependent telomere shortening varies on the basis of the replicative needs of the specific tissue ^138, 139^. They hypothesize that skeletal muscle (a minimally replicative cell type) may represent telomere length closer to the time of birth and that the gap between skeletal muscle and leukocyte (a rapidly dividing cell type) telomere length attrition could serve to aid understanding of the associations between telomere length and human disease with each patient, in effect, serving as their own control. The first such study testing this hypothesis showed that increased attrition of telomeres in leukocytes was associated with atherosclerotic cardiovascular disease ^140^.

### Single-nucleotide polymorphisms, telomere biology genes, and disease

**Figure 5.**
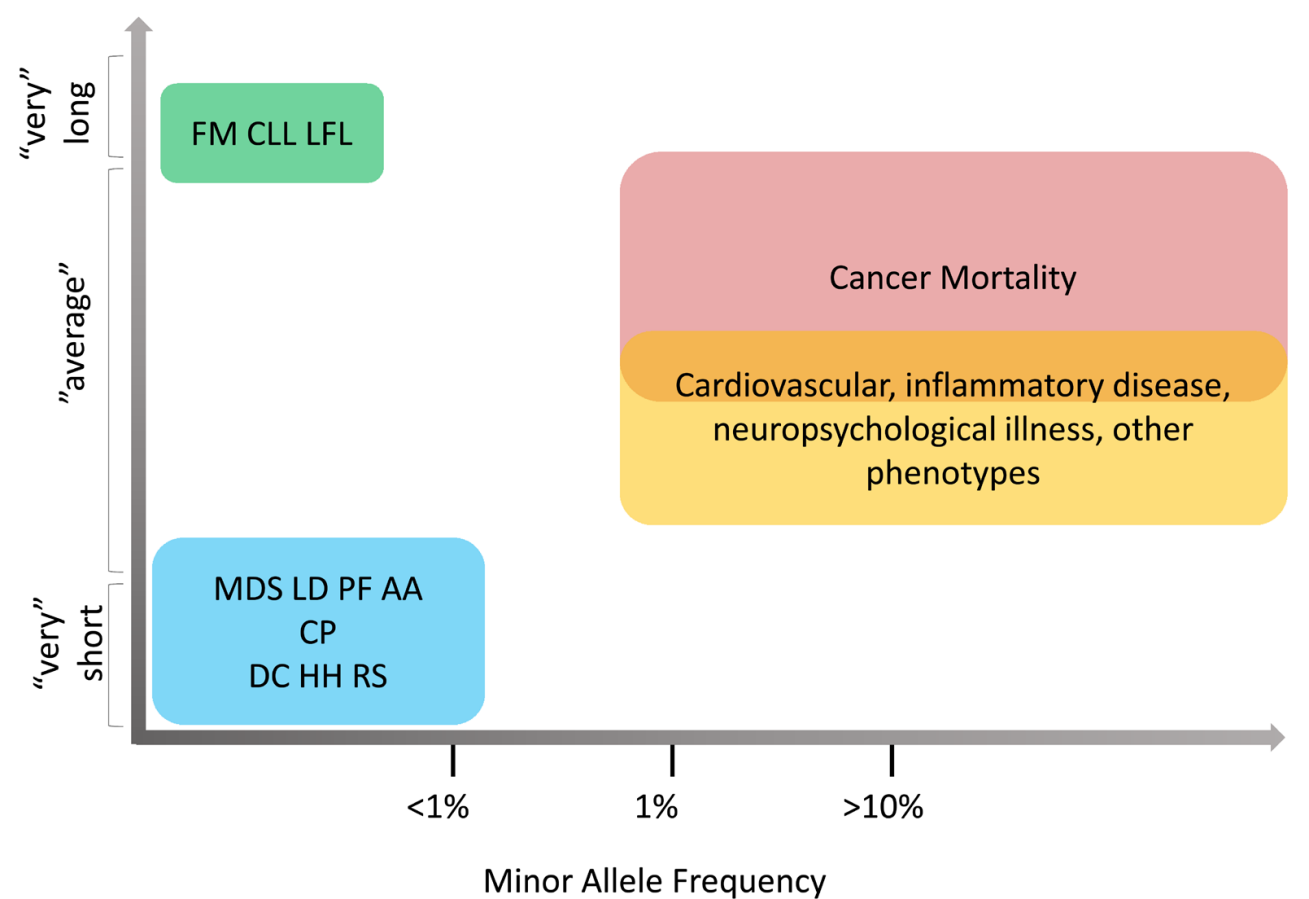
Relationship between telomere length, variant allele frequency, and human disease. The majority of genetic variants associated with common disease have a minor allele frequency (MAF) greater than 1% and telomeres in the “normal” range (that is, ** between the 1st and 99th percentiles for age). In contrast, genetic variants associated with rare and more highly penetrant disease are rare with MAF often much less than 1% and the extremes of telomere length. AA, aplastic anemia; CLL, chronic lymphocytic leukemia; CP, Coats plus; DC, dyskeratosis congenita; FM, familial melanoma; HH, Hoyeraal Hreidarsson syndrome; LD, liver disease; LFL, Li-Fraumeni-like syndrome; MDS, myelodysplastic syndrome; PF, pulmonary fibrosis; RS, Revesz syndrome.

The advent of genome-wide association studies (GWAS) opened the door to understanding associations between common genetic variants (that is, ** greater than 1% minor allele frequency, SNPs) and human disease or phenotypes ( Figure 5) (reviewed in 141). GWAS genotype hundreds of thousands of SNPs in thousands of cases and controls and use methods to fine-tune risk estimates through large-scale replication studies and polygenic risk score computation ^142^.

Numerous GWAS of cancer etiology have identified variants in telomere biology genes as being associated with cancer risk or outcomes. SNPs in the *TERT-CLPTM1L* locus on chromosome 5p15.33 are associated with multiple cancer types, including lung, pancreatic, breast, bladder, ovarian, prostate, and testicular germ cell cancers as well as glioma, melanoma, and non-melanoma skin cancers ^143– 146^. There are specific regions of this locus associated with different cancers, but these variants do not specifically encode deleterious coding alleles in *TERT*. They do, however, appear to be connected to telomere length through long-range regulation of this locus ^147^.

SNPs in *RTEL1* are associated with glioma in large GWAS of this rare brain cancer. The glioma-associated *RTEL1* SNPs are intronic, but functional studies have not yet been completed to understand their potential functions ^148– 152^. These findings are intriguing because patients with DC or HH due to autosomal recessive *RTEL1* mutations often have abnormal brain development in the form of cerebellar hypoplasia ^35– 37, 153^. Although the specific genetic loci are different, it is intriguing to speculate that there could be an important biological connection between these findings.

In addition to GWAS of cancer or other illnesses, several GWAS have been conducted to identify novel loci associated with telomere length. SNPs in known telomere biology genes, including *TERT*, *OBFC1* (encodes STN1), *CTC1*, *TERC*, *NAF1*, and *RTEL1*, as well as genes not previously known to be associated with telomere biology have been discovered ^154– 157^. These studies illustrate the complexities of telomere length regulation by showing that even common genetic variants, and especially combinations of common genetic variants, are associated with telomere length in the general population.

The existence of telomere length GWAS in various populations set the stage for even larger studies using Mendelian randomization methods in which telomere length–associated SNPs serve as surrogates for telomere length ^158^. One such study used nine telomere length–associated SNPs to create a telomere length surrogate score and found longer telomere length scores associated with lung adenocarcinoma but not the other cancers ^159^. Renal cell carcinoma, one of the cancers with reproducible telomere length association data, was also studied using nine telomere length surrogate SNPs and it was found that genetically longer telomeres were associated with renal cell carcinoma ^160^.

In 2017, a Mendelian randomization study of 16 telomere length–associated SNPs from 103 GWAS with summary data on 35 cancers and 48 non-neoplastic diseases found that genetically longer telomeres associated with elevated risk of many cancers, including glioma, ovarian cancer, lung cancer, neuroblastoma, bladder, skin, testicular germ cell cancer and kidney cancer, and endometrial cancer ^161^. That study also found an association between genetically shorter telomeres and the risk of interstitial lung disease, celiac disease, abdominal aortic aneurysm, and coronary heart disease but not of other inflammatory or psychiatric diseases ^161^.

Although several studies suggest that telomere length is associated with depression, one study using Mendelian randomization and three SNPs—one each in *TERT*, *TERC*, and *OBFC1*—as surrogates for telomere length in 67,000 individuals did not find an association between depression and genetically shorter telomeres ^162^. These investigators used the same three SNPs to investigate genetically predicted telomere length and risk of ischemic heart disease ^163^. They found small but statistically significant associations in a dataset of 60,837 ischemic heart disease cases compared with controls.

The studies briefly described above have generated a great deal of enthusiasm but are not without limitations. In many instances, qPCR was used to measure the telomere lengths in GWAS and this assay can be variable between studies. The sensitivity of the assay telomere length measurement and relatively small contributions of SNPs to telomere length should be considered in interpreting large-scale telomere length Mendelian randomization studies.

## The way forward

The connections between telomere biology and human disease are complex and myriad and require a multi-disciplinary approach to truly understand the clinically relevant data, important basic science questions, and implications of epidemiologic analyses ( Figure 1). As protectors of chromosome ends, telomeres are clearly integral to all aspects of cell biology. They are markers of biological aging and are regulated by a wide range of proteins.

Both very rare and very common germline genetic variants in telomere biology genes are associated with human disease, although the specific clinical phenotypes comprise a wide-ranging disease spectrum. Inheritance of telomere length inheritance and epigenetic regulation are also important aspects of telomere biology and should be incorporated into collaborative studies of rare and common telomere phenotypes. Additionally, optimization of telomere length measurement methods and improved understanding of environmental factors contributing to telomere biology will be essential in order to thoroughly understand these complexities. It is of the utmost importance for clinicians, epidemiologists, and basic scientists, all of whom study telomeres for a wide variety of different yet important reasons, to work together to build upon the expertise they each possess and incorporate that into improved understanding of telomere biology in human disease. This multi-disciplinary approach will enable the discovery of therapeutics and disease prevention modalities effective for patients with TBDs and for the general population.

## Abbreviations

AML, acute myeloid leukemia; CLL, chronic lymphocytic leukemia; DC, dyskeratosis congenita; GWAS, genome-wide association study; HH, Hoyeraal Hreidarsson syndrome; HNSCC, head and neck squamous cell carcinoma; MDS, myelodysplastic syndrome; qPCR, quantitative polymerase chain reaction; RS, Revesz syndrome; SNP, single-nucleotide polymorphism; TBD, telomere biology disorder.
